# Frequency of cholelithiasis in need of surgical or endoscopic treatment a decade or more after Roux-en-Y gastric bypass

**DOI:** 10.1007/s00464-022-09676-y

**Published:** 2022-10-06

**Authors:** Ingrid Belgau, Gjermund Johnsen, Hallvard Græslie, Ronald Mårvik, Siren Nymo, Kirsti Bjerkan, Åsne Hyldmo, Christian Klöckner, Bård Kulseng, Dag Hoff, Jorunn Sandvik

**Affiliations:** 1 Department of Clinical and Molecular Medicine, Faculty of Medicine and Health Sciences, NTNU, Trondheim, Norway; 2 Norwegian National Advisory Unit on Advanced Laparoscopic Surgery, Clinic of Surgery, St. Olav’s University Hospital, Trondheim, Norway; 3 Clinic of Surgery, Nord-Trøndelag Hospital Trust, Namsos Hospital, Namsos, Norway; 4 Faculty of Social Science and History, Volda University College, Volda, Norway; 5 Department of Surgery, Møre and Romsdal Hospital Trust, Ålesund Hospital, Ålesund, Norway; 6 Centre for Obesity Research, Clinic of Surgery, St. Olav’s University Hospital, Prinsesse Kristina’s Gate 3, 7030 Trondheim, Norway; 7 Department of Research and Innovation, Møre and Romsdal Hospital Trust, Ålesund, Norway

**Keywords:** RYGB, Cholelithiasis, Choledocholithiasis, BEA-ERCP, LA-ERCP, Post-bariatric abdominal pain

## Abstract

**Background:**

Symptomatic cholelithiasis requiring treatment is a known side effect after Roux-en-Y gastric bypass (RYGB), but reported rates vary greatly. The objectives for this study were to evaluate the long-term frequency of surgical or endoscopic treatment for symptomatic cholelithiasis 10–15 years after RYGB and its relation to self-reported abdominal pain.

**Methods:**

Observational data from 546 patients who underwent RYGB at public hospitals in Central Norway between March 2003 and December 2009 were analyzed.

**Results:**

Median follow-up was 11.5 (range 9.1–16.8) years. Sixty-five (11.9%) patients had undergone cholecystectomy prior to RYGB. Out of the 481 patients with intact gallbladder, 77 (16.0%) patients underwent cholecystectomy and six (1.2%) patients had treatment for choledocholithiasis during the observation period. Median time from RYGB to cholecystectomy or treatment of choledocholithiasis was 51 (range 1–160) and 109 (range 10–151) months, respectively. Female sex was associated with an increased risk of subsequent cholecystectomy [OR (95% CI) 2.88 (1.31–7.15)], *p* < 0.05. There was a higher frequency of self-reported abdominal pain at follow-up [OR (95% CI) 1.92 (1.25–2.93)] among patients who underwent cholecystectomy before or after RYGB.

**Conclusion:**

With a median follow-up of more than 11 years after RYGB, one in six patients with an intact gallbladder at time of RYGB underwent cholecystectomy, and 1.1% of the patients needed surgical or endoscopic treatment for choledocholithiasis. Patients with a history of cholecystectomy reported a higher frequency of abdominal pain.

Bariatric surgery is an established treatment for selected patients with severe obesity, and annually more than 660 000 people undergo bariatric surgery worldwide [1]. Roux-en-Y gastric bypass (RYGB) is a common bariatric procedure, shown to result in long-term significant weight loss and remission of comorbidities [2]. However, these benefits are accompanied by several possible side effects including abdominal pain and increased risk of treatment-requiring cholelithiasis [2, 3].

The incidence of cholelithiasis requiring cholecystectomy is known to be high after RYGB, but the reported rates vary [2–9]. This high incidence raises the question of prophylactic cholecystectomy (PC) or prophylactic treatment to reduce formation of cholelithiasis such as ursodeoxycholic acid (UDCA) [10, 11]. Furthermore, the treatment of choledocholithiasis following RYGB surgery can be challenging. It is not possible to perform regular endoscopic retrograde cholangiopancreatography (ERCP) due to the patient’s altered anatomy, necessitating the use of alternative techniques such as balloon enteroscopy-assisted ERCP (BEA-ERCP) or laparoscopy-assisted ERCP (LA-ERCP) [12–14].

Several factors may be related to the occurrence of cholelithiasis after RYGB surgery. Obesity by itself constitutes an increased risk as shown by Talseth et al. who found a twofold increase in frequency of cholecystectomy in people with obesity during a 15-year follow-up [15, 16]. In a Swedish study from 2009, the baseline cholecystectomy rate before RYGB was 3.4 times higher than the background population [3]. Rapid weight loss following RYGB further increases the risk of cholelithiasis [17]. Wanjura et al. reported an incidence ratio for cholecystectomy 6–12 months after RYGB at 3.5 compared to that before surgery [3]. This finding is coherent with other studies that have found an association between substantial and rapid weight loss and the incidence of postoperative cholelithiasis development [17, 18]. Gallbladder dysmotility, intestinal factors, metabolic conditions, and altered bile composition may also contribute to an increased risk of cholelithiasis after RYGB [19, 20]. Abdominal pain is a common symptom after RYGB and can be caused by a number of conditions [21, 22]. The relationship between abdominal pain and cholecystectomy has been less investigated.

The primary aim of this study was to evaluate the long-term frequency of surgical or endoscopic treatment for symptomatic cholelithiasis 10–15 years after RYGB. A secondary aim was to explore the relationship between self-reported abdominal pain and former cholecystectomy.

## Materials and methods

The Bariatric Surgery Observation Study (BAROBS) is a cross-sectional observational study supplemented by retrospective analysis of prospectively collected data from patients who underwent RYGB for severe obesity at three public hospitals in the Central Norway Regional Health Authority between 2003 and 2009. From a total of 930 patients who underwent RYGB in this period, and still were alive, 546 (58.7%) patients participated in the BAROBS study and underwent a clinical follow-up after 10–15 years. Whether the patients had known cholelithiasis prior to RYGB and whether they underwent cholecystectomy before or after the surgery, as well as any treatment for choledocholithiasis, were registered. Supplementary information about treatment of cholelithiasis was collected from the electronic patient record (EPR). Clinical follow-up was performed by doctors (both surgeons and non-surgeons), and patients were interviewed about the frequency of abdominal pain, categorized as daily, weekly, monthly, or seldom/never.

Adult patients with either BMI ≥ 40 kg/m^2^ or BMI ≥ 35 kg/m^2^ with obesity-related comorbidity, and without contraindications for bariatric surgery were offered RYGB. The surgical procedure was standardized between the hospitals and performed laparoscopically according to the Lönroth method [23]. There was no screening for asymptomatic cholelithiasis pre- or post-operatively, and UDCA was not given post-operatively.

Participation in the study was based on written informed consent. The study was approved by REK (Regional Committees for Medical and Health Research Ethics) in May 2018 (2017/1828/REK sør-øst).

### Statistical analysis

Statistical analyses were performed using IBM SPSS version 27 (SPSS Inc., Chicago, IL, USA) software. Categorical variables are presented as numbers and percentages, and continuous variables are presented as means ± SD if normally distributed, otherwise by median and range. The Pearson *χ*^2^ test was used for comparison of categorical variables with correction for multiple hypothesis testing. Student *t* test was performed to compare normally distributed continuous variables, and non-parametric test was performed to compare variables not normally distributed. Cox regression was used for time-to-event analysis, and in addition, logistic regression analyses were performed. *P* < 0.05 was considered statistically significant for all analyses.

## Results

We retrospectively evaluated 546 patients who underwent RYGB with a median follow-up of 11.5 (range 9.1–16.8) years. Four hundred and thirty-seven (80%) were females, and the mean age at RYGB surgery was 40.4 (± 9.0) years. The mean preoperative BMI was 44.3 (± 5.5) kg/m^2^. The mean nadir BMI was 29.6 (± 4.6) kg/m^2^, and the mean BMI at follow-up after 11.5 years was 35.0 (± 6.9) kg/m^2^. In this study, there were 17 patients (3.1%) included with a history of prior bariatric surgery. Apart from gender, there were no differences in baseline characteristics between patients with and without treatment for cholelithiasis (Table 1).

**Table 1:** Baseline characteristics

	Cholecystectomy before or after RYGB *n* = 142	No history of cholecystectomy *n* = 404	*p* value
Age Mean ± SD, years	40.8 ± 9.6	40.2 ± 8.8	ns
Male *n* (%)	8 (5.6)	101 (25.0)	< 0.001
Female *n* (%)	134 (94.4)	303 (75.0)	< 0.001
Duration of follow-up Mean ± SD, months	143.4 ± 18.5	140.7 ± 19.2	ns
Preoperative BMI Mean ± SD, kg/m^2^	44.3 ± 5.4	44.3 ± 5.6	ns
%TWL from RYGB to nadir Mean ± SD, %	32.5 ± 9.4	32.8 ± 8.7	ns
Nadir BMI after RYGB Mean ± SD, kg/m^2^	29.7 ± 4.8	29.6 ± 4.6	ns
BMI at follow-up Mean ± SD, kg/m^2^	34.3 ± 7.0	35.2 ± 6.9	ns

### Cholecystectomy

**Fig. 1:**
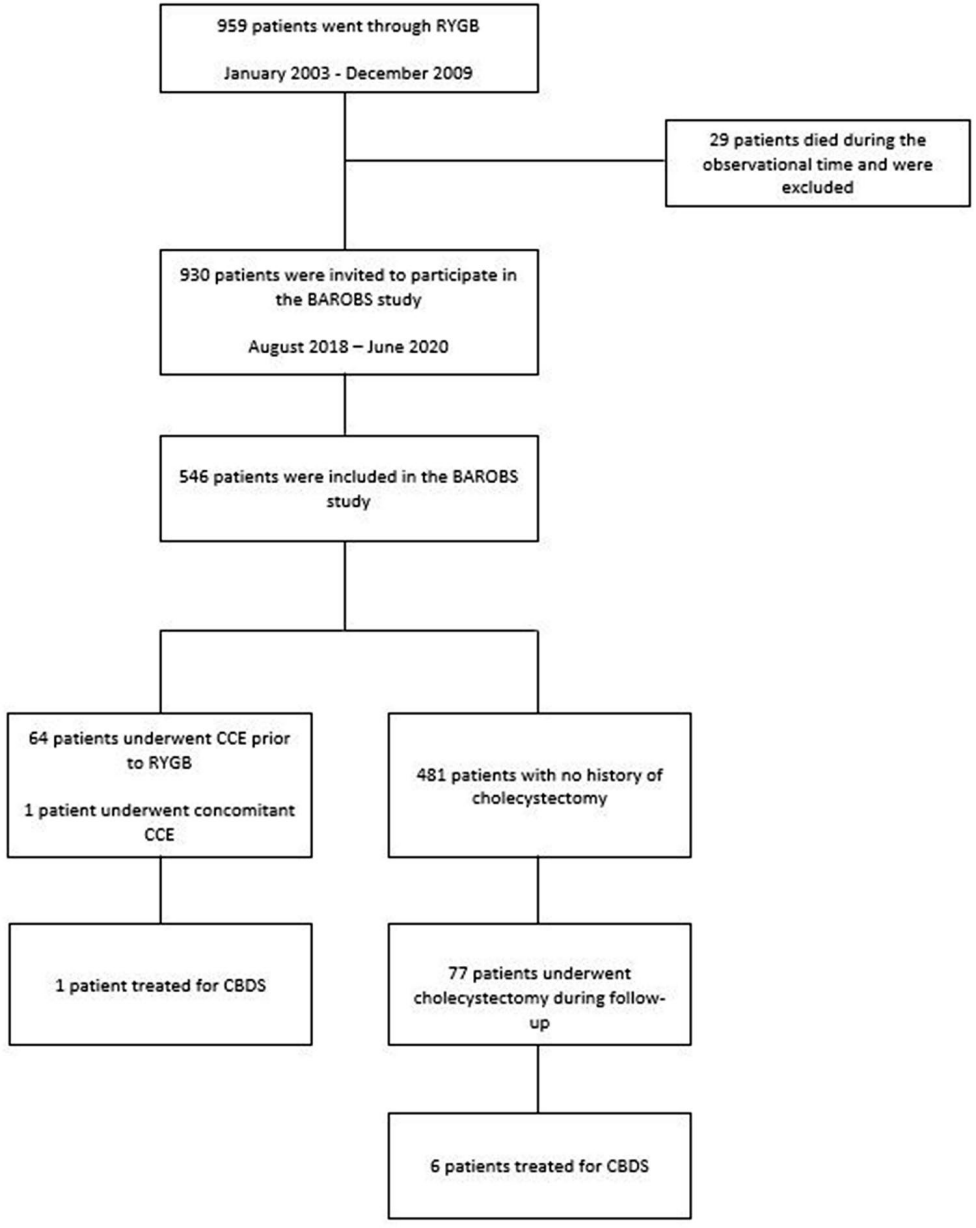
*RYGB* Roux-en-Y gastric bypass, *CBDS* common bile duct stone, *CCE* cholecystectomy

Sixty-five (11.9%) patients had undergone cholecystectomy prior to RYGB, including one concomitant cholecystectomy, and therefore excluded from further analysis (Fig. 1). Sixteen (2.9%) patients had known asymptomatic cholelithiasis prior to surgery.

**Fig. 2:**
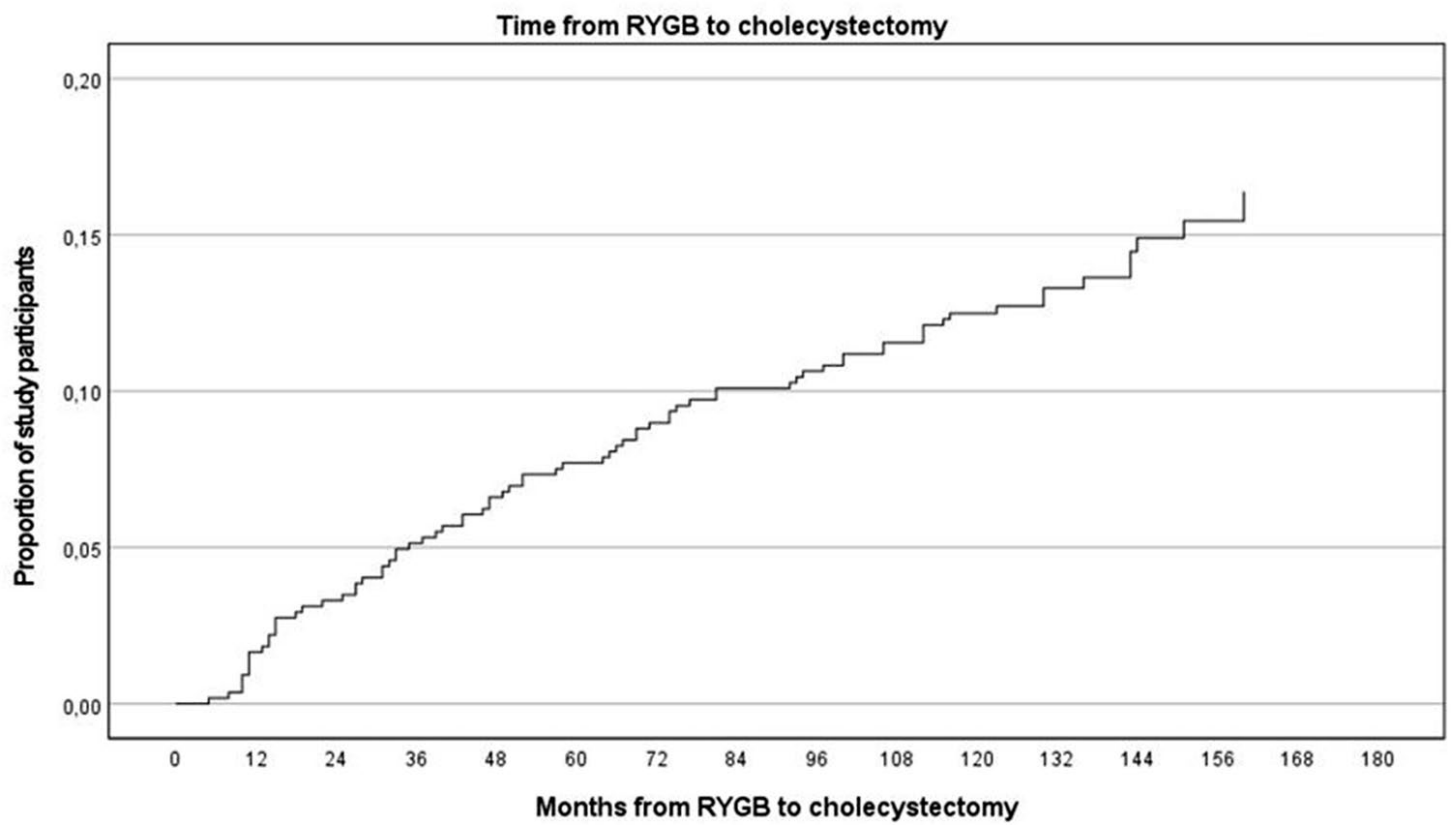
Cox regression. The proportion of study participants with no history of cholecystectomy before RYGB who had a cholecystectomy after RYGB

From 481 patients with intact gallbladder at time of RYGB, seventy-seven (16.0%) patients underwent cholecystectomy during the median follow-up of 138 (range 109–201) months. Forty-two (8.7%) patients had their gallbladder removed during the first 5 years after RYGB, and nine of these patients had their gallbladder removed during the first 12 months. Median time from RYGB to cholecystectomy was 51 (range 1–160) months (Fig. 2). The annual cholecystectomy frequency was nine (1.9%), nine (1.9%), ten (2.1%), and eight (1.7%) patients during the first, second, third, and fourth years following RYGB, then from two (0.4%) to seven (1.5%) patients annually during the fifth through thirteenth year.

Indications for cholecystectomy in patients with detected gallstones were classic biliary abdominal pain in 44 (57.1%) patients, non-classic biliary abdominal pain in 3 (3.9%) and cholecystitis in 20 (26.0%) patients. Ten (13.0%) patients underwent cholecystectomy due to choledocholithiasis related treatment or biliary pancreatitis. Cholecystectomy was more common among females, as 69 (18.6%) of 372 females compared to eight (7.3%) of 109 males had a cholecystectomy during the follow-up after RYGB. This gives female sex an increased risk of cholecystectomy over male sex (OR (95% CI) 2.88 (1.31–7.15), *p* < 0.05.

Of the 16 patients with known, asymptomatic cholelithiasis prior to surgery, six patients underwent cholecystectomy during the follow-up.

Logistic regression analyses, independent *t* tests or Pearson *χ*^2^ test did not reveal any association between subsequent cholecystectomy after RYGB and age, BMI at surgery, previous bariatric surgery, known asymptomatic cholelithiasis before surgery, percentage total weights loss (%TWL) from RYGB to nadir, or BMI at follow-up (Table 2).

**Table 2:** Analyzed risk factors for cholecystectomy after RYGB in patients with intact gallbladder at RYGB

	Cholecystectomy after RYGB	No cholecystectomy after RYGB	*p* values
Age at RYGB Mean (SD) years	39.0 (9.6)	40.2 (8.8)	0.274*
BMI at RYGB Mean (SD), kg/m^2^	44.1(5.4)	44.3 (5.6)	0.769*
BMI 11.5 years after RYGB Mean (SD), kg/m^2^	34.5 (7.0)	35.2 (6.9)	0.411*
%TWL from RYGB to nadir Mean(SD), %	32.8 (9.0)	32.8 (8.7)	0.997*
Sex			
Male	8/109	101/109	0.005^#^
Female	69/372	303/372	
Previous bariatric surgery			
Yes	2/13	11/13	1.0**
No	75/468	393/368	
Asymptomatic gallbladder stone prior to RYGB			
Yes	6/16	10/16	0.029**
No	71/465	394/465	

*Independent-samples *t* test

**Fisher's exact test, not significant after multiple hypothesis testing

^#^Pearson *χ*^2^ test, *p* < 0.05 after multiple hypothesis testing

### Choledocholithiasis

Six (1.2%) patients, three of them females, were in need of treatment for choledocholithiasis during the follow-up. Median time from RYGB to treatment for choledocholithiasis was 109 (range 10–151) months. One patient in the study had treatment for choledocholithiasis prior to RYGB.

BEA-ERCP was attempted in four patients. The procedure succeeded in two patients, while the other two patients went through subsequent LA-ERCP. One of these patients was further converted to an open transcholedochal bile duct exploration. Two patients had laparoscopic-assisted transcystic bile duct exploration.

Patients who were treated for choledocholithiasis had symptoms or signs of pancreatitis (*n* = 2), cholangitis (*n* = 1), cholecystitis (*n* = 2), or abdominal pain with cholestasis (*n* = 1).

### Abdominal Pain

The patients were asked whether and how often they experienced abdominal pain at the follow-up interview more than 10 years after RYGB. In the group with no history of cholecystectomy, 73 (18.2%) of 401 patients reported daily or weekly abdominal pain [OR (95% CI) 0.84 (0.72–0.97)]. In the group who had a cholecystectomy before or after RYGB, 41 (28.9%) of 142 patients reported daily or weekly abdominal pain [OR (95% CI) 1.53 (1.13–2.06)] (Table 3). No association between treatment for choledocholithiasis and reported abdominal pain was found.

**Table 3:** Association between self-reported abdominal pain and cholecystectomy before or after RYGB

	Cholecystectomy before or after RYGB *n* = 142	No history of cholecystectomy *n* = 404	*p* value^b^
Self-reported abdominal pain at interview: daily ^a^*n* = 543 *n* (%)	*n* = 25/142 (17.6)	*n* = 24/401 (6.0)	< 0.001*
Self-reported abdominal pain at interview: daily or weekly ^a^*n* = 543 *n* (%)	*n* = 41/142 (28.9)	*n* = 73/401 (18.2)	0.007*
Self-reported abdominal pain at interview: monthly ^a^*n* = 543 *n* (%)	*n* = 25/142 (17.6)	*n* = 37/401 (9.2)	0.007*
Self-reported abdominal pain at interview: seldom or never ^a^*n* = 543 *n* (%)	*n* = 76/142 (53.5)	*n* = 291/401 (72.6)	< 0.001*

^a^*N* number of respondents

^b^Pearson *χ*^2^ test

*Significant *p* value after correcting for multiple hypothesis testing

## Discussion

In the present study, one in six patients with an intact gallbladder at the time of RYGB developed postoperative symptomatic cholelithiasis and underwent cholecystectomy during the 10–15-year follow-up after RYGB. The frequency of cholecystectomy during the first 6 years was 10.3%, which is coherent with reported rates at 9.7–10.6% with a median follow-up of 49–70 months [4, 7]. However, several studies have reported considerably higher rates of cholelithiasis requiring cholecystectomy at 13.0–14.5% with an average follow-up of only 12–24 months [6, 8, 9]. The reason for these varying cholecystectomy rates following RYGB remains unknown.

Six out of sixteen patients with asymptomatic cholelithiasis at time of RYGB required cholecystectomy in the observation period. At the time of this study, the general practice was to avoid cholecystectomy in asymptomatic patients, as it would be more convenient to do cholecystectomy after weight loss, if needed. In the times of open bariatric surgery, however, prophylactic cholecystectomy was performed even in patients without cholelithiasis, but this practice is not recommended anymore.

Few studies have reported the long-term incidence of choledocholithiasis after RYGB. We found that 1.2% of the study participants needed treatment of choledocholithiasis during the follow-up. Haal et al. reported a 0.9% rate of choledocholithiasis during the first 24 postoperative months after RYGB [8]. Dirnberger et al. found a 1.2% rate of choledocholithiasis with a median follow-up of 63.3 months; however, these patients had either prophylactic cholecystectomy or prophylactic treatment with UDCA [24]. Even though the reported long-term incidence of choledocholithiasis is low, this condition can lead to serious illness and challenging treatment in post-RYGB patients.

Rapid and substantial weight loss after bariatric surgery is among the factors potentially influencing the risk of cholelithiasis. We did not find any association between higher %TWL to nadir and the frequency of cholecystectomy in this study. This is in line with findings in several studies showing no association between cholelithiasis formation and amount or rate of weight loss after bariatric surgery [6, 25, 26]. On the other hand, other studies have found a higher %TWL after bariatric surgery to be associated with an increased risk for cholecystectomy [8, 9, 27, 28].

Female gender was associated with an increased frequency of cholecystectomy in our study. As with weight loss, previous studies have shown ambiguous results, several studies find female gender to increase the risk of cholecystectomy after RYGB, while other studies find no such association [3–5, 7, 8, 18].

The use of UDCA for preventing cholelithiasis has shown promising results in several studies [2, 29–31]. A meta-analysis of randomized control trials from Fearon et al. concluded that UDCA reduces the risk of both symptomatic and asymptomatic cholelithiasis after bariatric surgery [32]. Contrarily, there are also studies showing no effect of UDCA on cholecystectomy rates [7]. In the study from Dirnberger et al., patients with preoperative known cholelithiasis were recommended prophylactic cholecystectomy and UDCA in case of no cholelithiasis on preoperative sonography. They found that 9.3% of the patients who were advised to use UDCA still went through subsequent cholecystectomy with a mean follow-up of 63.6 months [24]. These numbers are similar to the numbers in our study of patients without any prophylactic measures. However, a systematic review from Choi et al. concluded that prophylactic UDCA seems to be a reasonable preventive method following gastric surgery, decreasing the odds of needing subsequent cholecystectomy with nearly 80% [11].

The RYGB procedure leads to altered upper gastro-intestinal anatomy, making endoscopic approaches technically challenging. In our study, two patients out of four were successfully treated endoscopically with BEA-ERCP, and other techniques such as LA-ERCP had a high rate of success. Other studies support the finding that both LA-ERCP and BEA-ERCP have high success rates and are safe and reliable methods for managing choledocholithiasis after RYGB [12–14]. Tonnesen et al. found that LA-ERCP had a higher success rate as well as a higher incidence of adverse events than BEA-ERCP [12]. Supporting this, a meta-analysis from 2020 concluded that LA-ERCP had a significantly higher success rate compared to enteroscopy-assisted ERCP, but at the expense of longer procedural time and a higher rate of adverse events [33].

We found that patients with a history of cholecystectomy had a higher frequency of self-reported abdominal pain at follow-up a decade or more after RYGB. Patients who experience abdominal pain are more likely to be referred to abdominal imaging. Sandvik et al. found that 40% of the patients had one or more CT scans and 28% had one or more ultrasound scans due to abdominal pain during a mean follow-up of 8.3 years after RYGB [34]. Previous studies have shown that 24–48% patients will develop cholelithiasis after RYGB [9, 25, 26, 30, 35, 36], leading to a high probability of finding cholelithiasis during imaging with subsequent cholecystectomy.

The strengths of this study are the long-term follow-up and the ability to follow the patients across several institutions. The RYGB procedure was performed identically at the three participating hospitals, and the follow-up was standardized and in-office. There are limitations to this study. From a total of 930 patients, 546 patients (58.7%) participated in BAROBS. This is a high number compared to similar studies, but it may still lead to an overestimation or underestimation of incidence of gallstone disease. No systematic screening for cholelithiasis or abdominal pain was conducted prior to RYGB. Another limitation is the lack of a comparison group with patients who did not go through RYGB.

## Conclusion

The long-term incidence of symptomatic cholelithiasis requiring cholecystectomy after RYGB is high, but the incidence of complicated gallstone disease remains low. Patients with a history of cholecystectomy report a higher frequency of abdominal pain a decade or more after RYGB, which is an observation that needs to be investigated further.
